# An insight of pathological anatomy of urethral stricture and surgical manipulations for children suffered from hypospadias

**DOI:** 10.1186/s12894-023-01209-6

**Published:** 2023-03-18

**Authors:** Yuanhong Xiao

**Affiliations:** Department of Pediatric Surgery, Chinese PLA General Hospital, Nan Men Cang 5th, Dongcheng District, Beijing, 100700 China

**Keywords:** Child, Hypospadias, Pathology, Surgery, Urethral stricture

## Abstract

**Background:**

Manipulation of urethral stricture is difficult and challenging. Accurate analysis and evaluation of the pathological circumstances of narrowed urethra and surrounding tissues were important for cure. The detailed descriptions of anatomic pathology of urethral stricture are rare. An insight of the pathological anatomy of the strictured urethra and the corresponding corrections is essential for an ultimate cure. The aim of the study is to interpret the pathological anatomies of the strictured urethra and the corresponding surgical manipulations.

**Methods:**

From April 2007 to April 2020, eight boys who suffered from postoperative urethral stricture for hypospadias correction were studied retrospectively. The pathological anatomies of the strictured urethra were described and the corresponding surgical manipulations were analyzed.

**Results:**

All eight patients were eventually cured through surgery. The patient age was 2.58–11 years old (mean value of 4.73). The therapeutic duration was 12–130 months (mean value of 47.75). The surgical manipulation was 1–9 times (mean value of 4.5). Curative follow-up was 8–138 months (mean value of 77.75). They were all applied with flap tubularization for their primary urethroplasty.

**Conclusions:**

Based on the principal contradiction of the pathological anatomies of the urethral stricture, one-stage or staged surgical manipulations can be applied. After stricture was resolved, refining techniques of Mathieu, TIP, modified Duckett, glanular reconstruction and et al. can be used. Although it was tiring, utilizing the ventral or dorsal penile flap with relatively good blood supply and flexibility to reconstruct the urethra is possible and successful. To reconstruct an anatomically normal urethral tract should be pursued in the primary and consequential procedures for hypospadias and urethral stricture.

## Background

Urethral stricture is the second common surgical complication for hypospadias, which is strongly connected with the primary operation, especially the flap tubularization urethroplasty [1–5]. Manipulation of urethral stricture is difficult and challenging especially after multiple procedures with local scars and sparse tissues available to be used [6–8]. Majority of the urethral strictures should be corrected through surgery [9–13]. Substitution tissues for urethral reconstruction from distal sites can be applied with a risk of additional complications [14–20]. In the literature, the pathologic descriptions of urethral stricture confined to mention of the anatomic portion, such as shaft or bulb of the penile. About the meticulous insight description and detailed pathologic presentations of the urethral stricture have not been proposed [21–25], which we think valuable for the surgical manipulations of urethral stricture. In this article, through eight patients’ successful surgical treatments, we tried to respectively describe and analyze the pathological anatomy of the strictured urethra and its surgical manipulations.

## Methods

### General data

From April 2007 to April 2020, eight boys suffered from urethral stricture after hypospadias corrections were included in this study. Flap tubularization urethroplasty was the primary procedure for all of them. The age ranged from 2.58 years old to 11 years old, with a mean age of 4.73 years old (Table 1 General data and results of 8 patients). The pathological anatomies of the strictured urethra was described and the corresponding surgical manipulations were analyzed (Table 2 Pathological anatomies of the urethral stricture and surgical manipulations).

**Table 1:** General data and results of 8 patients

Cases	Age of urethral stricture diagnosed (Y)	Frequency of surgical manipulations for urethral stricture	Surgical duration for hypospadias from initial therapy to cure (M)	Follow-up period (M)
1	4	1	12	111
2	4.58	2	21	136
3	4	1	14	137
4	4.5	5	18	138
5	11	7	130	38
6	4.17	8	89	8
7	3	9	65	45
8	2.58	3	33	9

**Table 2:** Pathological anatomies of the urethral stricture and surgical manipulations

Cases	Pathological anatomy of urethral stricture	Surgical manipulations
1	Coronal meatal stenosis Proximal tiny fistula	Heineke–Mikulicz+Mathieu+Glanular reconstruction Simple fistula repair
2	Fossa-proximal meatal stenosis Proximal fistula	Heineke–Mikulicz Flap advancement
3	Segmental stricture of penilel shaft urethra with elastic adjacent corpus spongiosum	Heineke–Mikulicz+Corpus spongiosum approximating anastomosis
4	Segmental stricture with inelastic adjacent corpus spongiosum of penilel shaft Sub-coronal retreated meatus Tiny fistula	Heineke–Mikulicz+Catheter supporting TIP+Glanular reconstruction Simple repair
5	Severely stenotic coronal meatus with epidermis-injured glanular plate Huge diverticulum with good elasticity Pinhole fistula Small glans Peno-scrotal transposition	Stage I: Meatal stenosis elimination; Resuming and widening glanular plate; Diverticulum elimination; Snodgrass; Simple fistula repair Stage II: Mathieu+Glanular reconstruction+Scrotoplasty
6	Slightly stenotic meatus Shabby pouch-like Diverticulum without elasticity Fistula Small glans Chordee recurence Peno-scrotal transposition Fistula recurrence	Stage I: Diverticulum elimination; Mathieu+Glanular reconstruction Stage II: Duckett+Scrotoplasty Serial fistula repairs: Turn-over rectangle flip-flap; Y-shaped incision flap advancement
7	Complete aplasia of the whole anterior urethra originally Urethral opening stricture Urethral stricture Proximal fistula Proximal urethral dilation Chordee recurrence Coronal meatus	Stage I: Catheter indwelling of the strictured urethra+Proximal urethral-ostomy Stage II: Rectangle flip-flap for ostomy covering Subsequent repairs: Tunica albuginea plication+TIP+Glanular reconstruction
8	Complete aplasia of the whole anterior urethra originally Urethral opening stricture Urethral stricture Proximal fistula Proximal urethral dilation Peno-scrotal transposition	Stage I: TIP+Scrotoplasty+Heineke–Mikulicz Stage II: Mathieu+Glanular reconstruction

*Pathologic anatomy of the urethral stricture and surgical manipulations* (Table 2 Pathological anatomy of the urethral stricture and surgical manipulations).

For cases with urethral opening stenosis and proximal fistula, Heineke–Mikulicz technique was applied to release opening stenosis. Mathieu combined with glanular reconstruction was preformed for a coronal meatus (Case 1). For a more distal meatus approximating to fossa, Heineke–Mikulicz technique was enough (Case 2). Simple repair or flap advancement for fistula closure was followed subsequently according to the size of the fistula.

For cases of segmental urethral stricture of the penile shaft, the manipulations were different according to the adjacent corpus apongiosum circumstances. When the segmental stricture occurred within an elastic corpus spongiosum, one-stage Heineke–Mikulicz technique combined with corpus spongiosum approximating anastomosis can solve the problem (Case 3). While when the inelastic corpus spongiosum was highlighted by a significant segmental stricture, after Heineke–Mikulicz for stricture releasing, three months catheter supporting was advisable. A subcoronal meatus was predictable for his primary procedure with a dorsal longitudinal penile flap, wherein the length and width of the tubularization flap was 3.5 cm and 1.5 cm respectively with a narrow blood supply. TIP combined with glannular reconstruction was suitable. Simple repair was cured for a residual coronal tiny fistula (Case 4).

**Fig. 1:**
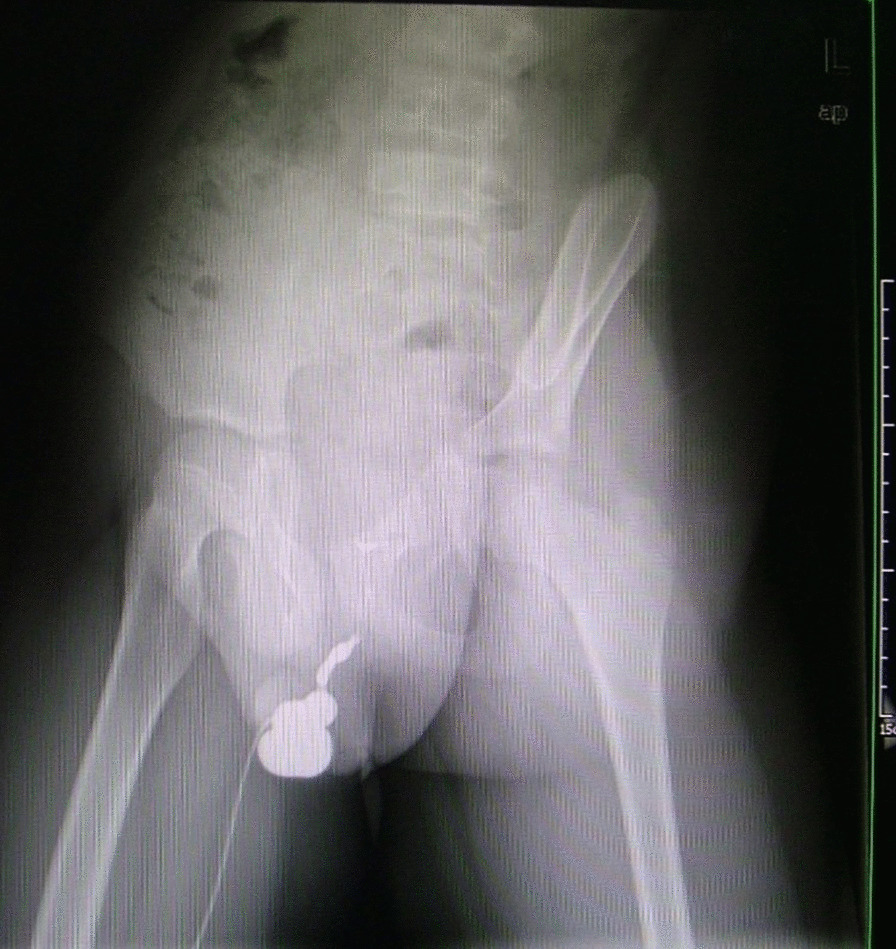
Urinary cystourethrography showed the huge diverticulum of anterior urethra

**Fig. 2:**
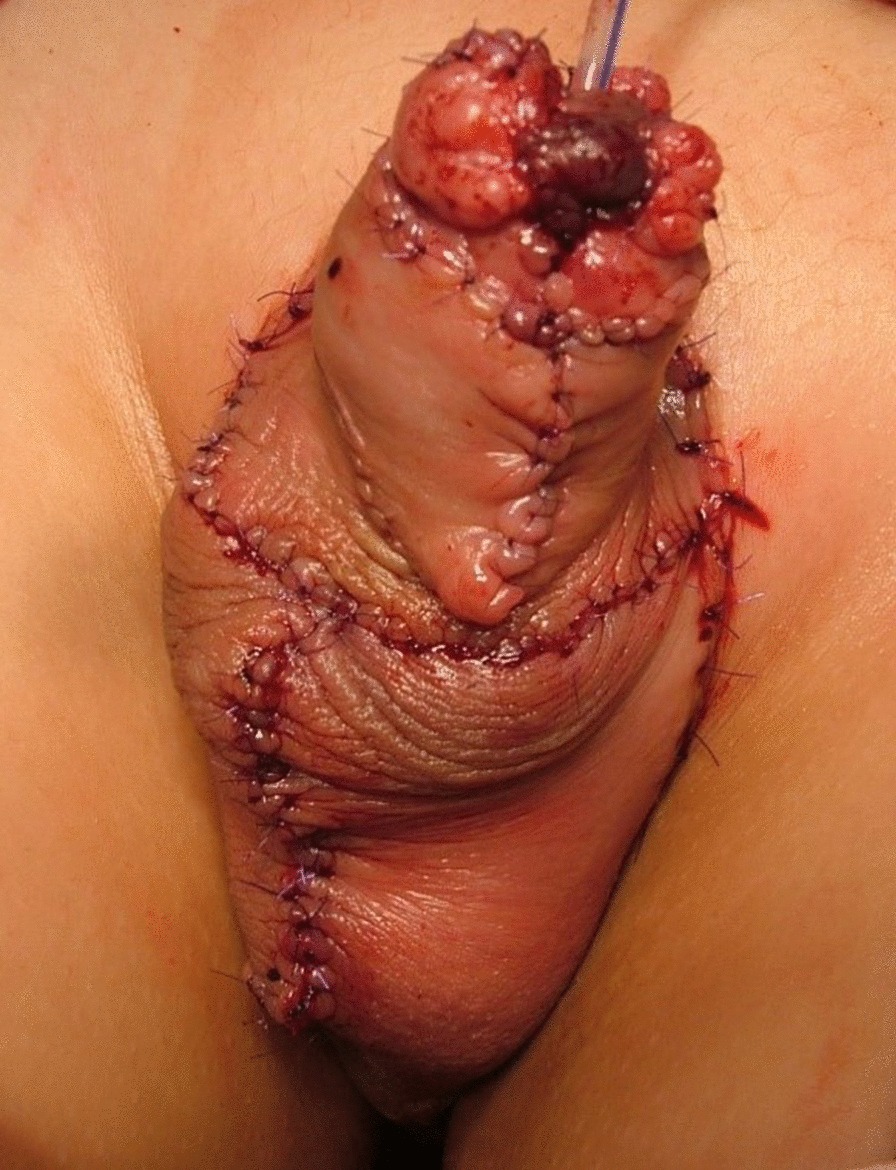
Completion of procedures of urethroplasty and scrotoplasty for urethral stricture and peno-scrotal transposition

Case 5 presented with a severely narrowed meatus like a horizontal crack of 0.3 cm × 0.1 cm with white color. The glans was small significantly disproportionate to the diameter of the penile. Glans urethral plate distal to the crack meatus was injured broadly due to the infected meatus and repeated home dilatation with a sound. Some epithelium of the plate was missing. Proximal to the severely stenotic meatus, a huge cucurbit-shaped diverticulum of 3.0 cm × 0.5 cm formed (Fig. 1 Urinary cystourethrography showed the huge diverticulum of anterior urethra). The diverticulum wall was vascularized well with a good flexibility. There were muddy stones inside of the diverticulum. A needle hole fistula was forced to form at the bottom of the diverticulum for urine oozing during painstaking urination. Before admission to our hospital, he had endured 5 surgical manipulations and twice urethral dilatation by doctor and sequential urethral dilatation by himself at home. He even endured an episode of acute epididymitis of his right scrotum after the fourth procedure of urethroplasty. A ventral long incision from the stenotic meatus through the diverticulum was made. Redundant diverticulum tissue was tailored. Horizontal suture was made to resume and widen the injured glanular plate. Snodgrass procedure was used for penile urethroplasty with a coronal meatus and unimpeded urinary flow. Secondly, Mathieu procedure and glanular reconstruction simultaneously with scrotoplasty provided him a normal fossa meatus and a normal arrangement of penile-scrotum position. He had been followed up for 38 months with great satisfaction (Fig. 2 Appearance of penile and scrotum after corrections of urethral stricture and peno-scrotal transposition). Case 6 presented with a small glans, slightly stenotic meatus, fistula at the root of the penile, diverticulum proximal to the fistula. The whole urethral tissue was just like an inelastic pouch. When dissected, the left lateral wall of the diverticulum retracted to the depth and a disastrous state of its inner side came into view. Erosive mucosa with granular protuberance diffusedly distributed on the inelastic surface of the pouch. Firstly, pouch was reconditioned, followed by Mathieu glanular urethroplasty and glanular reconstruction. Secondly, the nature of fistula and slight penile chordee was realized as a result of the crippled urehtral tract, for there was only a vulnerably slight connection of the dorsal wall of the so-called fistula. When this connection was incised, the proximal and distal end of the inelastic urethral tissue separated immediately to form a 2 cm gap and the chordee disappeared also. Modified Duckett procedure was used to substitue the urethral defect. Simultaneous scrotoplasty was used for correction of penile-scrotum transposition. Serial fistula repairs including turn-over rectangle flip-flap, Y-shaped incision flap advancement, mattress suture techniques were applied for the subsequnet emerging of fistula.

When urethral opening stricture, urethral stricture accompanied with proximal fistula and urethral dilation were co-existed, manipulations became very challenging due to more severe scars of the urethral tract and its surrounding tissues. The tissues were extensively edematous, thickening, stiffening with an indistinct border. Cases 7 and 8 presented with this pathological characteristics. Interestingly, they both presented with a complete aplasia of the corpus spongiosum. For case 7, he was applied urethral tubularization using dorsal longitudinal penile flap of 3.0 cm × 0.8 cm as the primary urethroplasty. Duplay procedure with a 0.8 cm wide U-shape peritoneal flap was tubularized to connect with the flap-tube. The meatus retreated to the coronal site postoperatively. Scar contraction contributed to chordee developement. Six manipulations including dilatation, catheter-indwelling, diverticulum flap transposition for urethral substitution and the ultimate suprapubic cystostomy resulted in an incomplete resolution. Acute epididymitis of his right scrotum occurred on the most severely stenotic state. Urethral and paraurethral scar resection and urethral-ostomy with a silica supporting catheter maintaining in the distal narrowed urethral lumen for two years was manipulated, which was considered the turning point for an ultimate resolution of stricture. Secondly, rectangle flip flap of penile was turned over for urethral-ostomy coverage. Coronal meatus and chordee recurence was resolved through tunica albuginea plication, TIP and glanular reconstruction. He had been followed up for 45 months without later complications. For case 8, Duckett accompanied with Duplay procedures were applied primarily. The transverse tubularized inner prepuce island flap with a size of 5.0 cm × 1.2 cm was united with a V-shape perineal flap. His perineal skin was congenitally coarse with a compromised extensibility. Perioperative fistula and dysuria occurred. Second look discovered urethral and surrounding tissues stiffness with strong adhesion. Widely and highly edematous and inelastic tissue made it difficult to distinguish the tissue borders. The meatus appeared almost atresia with a layer of thin film. Urethral lumen distal to the fistula was constrictive. The proximal anastomotic site presented with protuberant granular scar. Heineke–Mikulicz technique with absorbable 7-0 PDS II was used to resolve anastomotic stenosis. TIP procedure was performed with a coronal meatus for prevention of the meatal stenosis. Foley catheter was maintained for three months in case of stenosis recurrence. Two years later when the scar was softened, he was applied the third operation. Mathieu procedure together with glanular reconstruction was made to abtain an integrated urethral tract and fossa meatus. He had been followed up for 9 months without complications.

### Criteria of cure

Micturition was smooth without dysuria. The penile was straight without chordee. The meatus sited at the glans fossa. No fistula was residual.

## Results

All eight patients had been cured with a follow-up of 8 months to 138 months (mean value of 77.75). Hypoplasia or aplasia of the corpus spongiosum was their originally common features. Tubularization flap urethroplasty was their primary procedure. Even some similar pathological characteristics may be showed among the urethral stricture cases, individual one stage or staged manipulations for urethral stricture should be considered according to the principal pathological anatomic contributions (Table 2 Pathological anatomies of the urethral stricture and surgical manipulations). The therapeutic duration was 12 to 130 months (mean value of 47.75). The surgical manipulation was 1 to 9 times (mean value of 4.5) (Table 1 General data and results of 8 patients).

## Discussion

Severely hypoplasia or even aplasia of corpus spongiosum is a predictably original pathological specialty of hypospadias children who are inclined to develope urethral stricture after urethroplasty. The optimal choice of the primary urethroplasty and technical refinements are vital factors for a successful result [26–30]. We prefer Duckett transverse vascularized inner prepuce flap as the primary procedure. This technique ascertains an adequate vascularization of the flap. A long dorsal penile flap supplied by a relatively narrow vascular pedicle is inclined to contract. For our group, the two patients applying the dorsal penile transferred flap procedure, length to width ratios of their longitudinal flap is 2.33:1 and 3.75:1. The ratios are all over 2:1, which should be the limited value for an intrinsic blood supply for a primary longitudinal flap. We think that it is the ischemic contracture that result in the urethral stricture. Contracture and limited length make it difficult to obtain an orthotopic meatus and chordee is inclined to emerge. It is important to understand and follow the design principle of original procedures for hypospadias. To reconstruct an anatomically normal urethral tract should be the goal that we pursue all the time. Duplay V type perineal flap gave us a lesson for urethral stricture development. We emphasize that an adequate U type perineal flap tubularization is the rule of Duplay procedure. Fortunately, although it is tiring for the therapeutic process of urethral stricture utilizing the flap in situ, the ultimate result is good. We need time and patience for urethral stricture therapy.

The management principle of the urethral stricture should be based on its individual pathological anatomy. The principal contradiction of urethral pathological changes should be resolved first. For case 1 and 2, urethral opening stricture is the main pathological variation. Effective enlargement of the urethral opening and enough space for accommodation of glanular urethra are two important factors. So, Heineke–Mikulicz technique for the enlargement of the meatus and advancement of the dorsal wall of the urethral meatus, glanular wings formation for an adequate space for glanular urethra, Mathieu flip-flap for glanular urethroplasty are all requested techniques. Moreover, considering the compromised blood supply and inelasticity of the penile skin, simple fistula repair should be replaced by flap advancement or a rectangle flap to avoid overlap of anastomoses except for a very tiny one. For case 3 and 4, when the adjacent corpus spongiosum is elastic, Heineke–Mikulicz technique for the narrowed urehral portion with a routine catheter-indwelling period is enough. For a narrowed ring of an inelastic urethral tract, it is necessary to sustain the catheter supporting for three months. For case 5, the severely narrowed meatus is the principal contradiction of pathology. Staged procedure should be arranged. Stenotic meatus releasing, diverticulum elimination and glanular urethral plate reconstruction and widening (due to a small glans) will be the first step. Then Mathieu procedure and glanular reconstruction will be applied for an integrate glanular urethroplasty and an orthotopic meatus. For case 6, an inelastic urethral pouch is the pathological nature. Fistula is an appearance of urethral defect. Slight narrow of the meatus will magnify the lesion of the crippled urethral flap. The unhealthy urethral flap will not grow synchronously with the corpus cavernosum. Repeated fistula or chordee will emerge. Ventral and dorsal penile skins for fistula repair and modified Duckett procedure for urethral defect can be used. For case 7 and 8, when a longer urethral stricture segment including meatus is emerging, it is unhesitating to obtain a staged manipulation. For the patient experienced multiple procedures with severe scars of urethral tract and surrounding tissues, urethral ostomy and catheter indwelling to support the distal urethral lumen should be performed. We recommended 2 years period for scar softening and distal urethral catheter supporting before adjacent penile flip-flap covering the stoma. When TIP procedure or Mathieu procedure will be chosen for distal urethroplasty, more emphasis should be focused on the refined techniques of glanular reconstruction and glanular urethroplasty to provide an adequate space for glanular urethral accommodation. If the patient’s urethral and surrounding tissues are not so terrible, TIP procedure can be tried firstly. A coronal meatus is recommended for this stage in case of stenosis, and the supporting catheter is left for three months. Again, an additional two years interval should be waited for the scars softening. Then Mathieu and glanular reconstruction will accomplish the last success of an integrate urethra with a normal function.

## Conclusions

Based on the principal contradiction of the pathological anatomies of the urethral stricture, one-stage or staged surgical manipulations can be applied individually. After stricture is resolved, refining techniques of Mathieu, TIP, modified Duckett, glanular reconstruction procedures and et al. can be used. Although it is tiring, utilizing the ventral or dorsal penile flap with relatively good blood supply and flexibility to reconstruct the urethra is possible and successful. To reconstruct an anatomically normal urethral tract should be pursued in the primary and consequential procedures for hypospadias and urethral stricture.

## Data Availability

All data generated or analyzed during this study are included in the patients’ medical record data and patients’ photography data collected by the author. All the data supporting the article findings can be found in the data repositories of the author and the medical records of the patients which can be showed anytime if necessarily to ascertain the truth and science of this article based-on. The article is not related to the identifying/confidential patient data. All the contents and photographs in the article can be discussed and analyzed only for scientific and academic studies, not for other reasons. The author does not wish to share the original data with others.

## Abbreviations

Duplay procedure: Perineal U-type flap urethroplasty
Glanular reconstruction: Glanular wings formation together with Heineke–Mikulicz technique
Heineke–Mikulicz technique: Vertical incision and horizontal closure of the incised urethral tissue
Mathieu: Perimeatal flip-flap urethroplasty
Duckett procedure: Transverse vascularized inner prepuce flap urethroplasty
Modified Duckett procedure: Transverse dorsal penile island flap urethroplasty
Snodgrass procedure: Tubularized plate urethroplasty
Scrotoplasty: Bilateral rotational advancement flaps with relocation of the scrotal compartment
TIP procedure: Tubularized incised plate urethroplasty
